# Behaviour of Non-O157 STEC and Atypical EPEC during the Manufacturing and Ripening of Raw Milk Cheese

**DOI:** 10.3390/foods9091215

**Published:** 2020-09-01

**Authors:** Edson A. Rios, Juliana Ramos-Pereira, Jesús A. Santos, Teresa M. López-Díaz, Andrés Otero, Jose M. Rodríguez-Calleja

**Affiliations:** 1 National Institute of Science and Technology for the Dairy Production Chain (INCT-Leite), Paraná 10011, Brazil; edsonrios1@hotmail.com (E.A.R.); jramp@unileon.es (J.R.-P.); 2 Department of Food Hygiene and Food Technology, Veterinary Faculty, University of León, 24071 León, Spain; j.santos@unileon.es (J.A.S.); teresa.lopez@unileon.es (T.M.L.-D.); aotec@unileon.es (A.O.)

**Keywords:** diarrhoeagenic *E. coli*, raw cow’s milk, cheese processing, atypical EPEC, non-O157 STEC, behaviour

## Abstract

This study was carried out to assess the survival of Shiga toxin-producing *E. coli* (STEC) and atypical enteropathogenic *Escherichia coli* (aEPEC) during the traditional manufacturing and ripening of Spanish hard cheese from raw cow’s milk. Milk samples were spiked with up to 3.1–3.5 log cfu/mL of one strain of STEC (O140:H32 serotype) and one of aEPEC (serotype O25:H2). The first steps of cheesemaking allow for a STEC and aEPEC increase of more than 1 log cfu/mL (up to 4.74 log cfu/g and 4.55 log cfu/g, respectively). After cheese pressing, a steady reduction of both populations was observed, with the STEC strain being more sensitive. The studied pathogenic *E. coli* populations decreased by 1.32 log cfu/g in STEC and 0.59 log cfu/g in aEPEC in cheese ripened during a minimum period of 60 d. Therefore, a moderate contamination by these diarrhoeagenic *E. coli* pathotypes, in particular, with aEPEC, on cheese manufactured from raw milk may not be totally controlled through the cheesemaking process and during a maturation of 90 d. These findings remark the importance of improvement in bacteriological quality of raw milk and cross-contamination prevention with diarrhoeagenic *E. coli* in the dairy industry.

## 1. Introduction

Shiga toxin/verocytotoxin-producing *Escherichia coli* (STEC) represent one of the most common causes of foodborne illness in the world. Ruminants are thought to be the main reservoir of STEC [1] and their related foodstuffs have been associated with human outbreaks [2,3]. For 2018, 30 European Union (EU) countries reported 8658 confirmed cases of infection with STEC, and the overall notification rate increased by 41% in comparison with the stable period from 2014 to 2017 according to the last annual epidemiological report in EU [4].

Although the most commonly reported STEC serogroup in confirmed cases of human STEC infections was O157, the proportion of non-O157 serogroups has been increasing over the years and non-O157 serogroups have been involved in many haemolytic uremic syndrome (HUS) cases, suggesting an increased risk of acute infections by this group of bacteria [4]. These non-O157 serogroups were also responsible for 68.1% of STEC infections in the EU [5]. A similar increment of the non-O157 serogroups was observed in the USA, where the Centers for Disease Control and Prevention reported a total of 1272 cases of STEC infections, 60.2% caused in 2015 by non-O157 strains [6].

Enteropathogenic *E. coli* (EPEC) is a foodborne diarrhoeagenic *E. coli* responsible for deaths in children with diarrhoea worldwide [7]. EPEC is classified into typical (tEPEC) and atypical (aEPEC) strains based on the presence of the virulence plasmid known as EPEC adherence factor plasmid (pEAF), present in tEPEC and absent in aEPEC. Humans are the main reservoir of tEPEC, while aEPEC may also have as reservoir a wide variety of domestic and food-producing animals [8,9,10]. It is considered that aEPEC is an emerging pathogen and more prevalent than tEPEC in developed and developing countries [11].

Cheese is one of the most popular foods all over the world with very interesting nutritional properties. It is produced from milk and other non-dairy ingredients, such as lactic acid bacteria and enzymes. Global cheese consumption has increased considerably in the last decade. Approximately one-third of the total milk production in the EU is transformed into different types of cheeses. Some of these are made from unpasteurised milk. Foodborne infections linked to cheese consumption are also associated with cheeses made with unpasteurised milk. Risk of illness by pathogenic bacteria considerably increases in cheese ready to be commercialised. The main bacterial species involved in foodborne diseases associated with cheese consumption include *Escherichia coli*, among other Gram-negative and Gram-positive bacteria [12].

STEC and aEPEC have been usually isolated from animals used for food production, mainly in cattle [8,13,14,15]. Furthermore, milk can be contaminated by pathogenic microorganisms during the milking or the manufacturing of dairy products, in particular if mandatory good manufacturing practices were not followed [16]. Nevertheless, the presence of STEC and aEPEC in milk and dairy products, such as cheese manufactured from raw milk, has been frequently reported [10,17,18,19,20]. Nowadays, there is an significant number of works in the scientific literature studying the behaviour of STEC during cheese manufacturing, most of them focused on the O157:H7 serotype, neglecting the importance of non-O157 serogroups [21,22,23,24,25]. However, strains of non-O157 serotypes found in milk and dairy products [26,27] suggest that they could be as resistant to inhibition factors of food processing as the O157 serogroup. While information concerning other STEC serogroups is scarce, there is no information about the fate of aEPEC strains during processing of raw milk cheese [2].

Therefore, the aim of this study was to evaluate the behaviour of two strains of pathogenic *E. coli*—non-O157 STEC and aEPEC—isolated from milk, during the manufacturing and ripening of raw cow’s milk cheese.

## 2. Materials and Methods

### 2.1. Bacterial Strains

The studied strains of *E. coli* were STEC MK116C19 (O140:H32 serotype) and aEPEC MK127C9 (O25:H2 serotype), both isolated from cows’ milk [8]. The strains were cultured in Tryptone Soya Broth with 0.6% of Yeast Extract (TSB-YE, Oxoid, Basingstoke, United Kingdom) at 37 °C for 24 h from stock cultures maintained at −40 °C in TSB containing 20% (v/v) glycerol (Panreac, Barcelona, Spain). Thereafter, they were plated on Sorbitol MacConkey Agar plates (SMAC, Oxoid), which were stored overnight at 37 °C. A single colony of each strain was transferred into 30 mL flasks of TSB-YE and incubated at 37 °C for 6 h under continuous shaking (150 rpm). Aliquots (30 μL) of each resulting culture in stationary phase were inoculated into 30 mL TSB-YE and incubated at 37 °C for 18 h under shaking (150 rpm) to prepare working cultures.

The two tested strains were chosen among 25 strains which demonstrated good ability to adapt to intrinsic conditions of cheese (pH and NaCl). To carry out this assessment, 16 STEC and 19 aEPEC strains were studied in vitro for monitoring their growth ability in different pH and NaCl concentrations. Strains were inoculated with ~100 cfu/mL from working cultures obtained following the procedure described above. Aliquots (400 μL) were dispensed into microtiter plate wells and absorbance at 580 nm was measured for 48 h using a Bioscreen C instrument fitted with Biolink software (Labsystems Co., Helsinki, Finland). Growth rates were determined at 30 and 37 °C with NaCl concentrations between 0.5 and 6.0% and initial pH values ranging from 3.5 to 6.5.

### 2.2. Collection of Raw Cow’s Milk and Experimental Design

Raw cow’s milk was obtained from a bulk tank and transported at 4 °C to the laboratory within 3 h. Milk was tested for the detection of *stx* and *eae* genes by PCR using the primers and conditions reported elsewhere [28,29,30].

Two types of cheeses were manufactured: (a) control cheeses from raw cow’s milk and (b) spiked cheeses contaminated with a mixture of *E. coli* strains STEC MK111C9 and aEPEC MK127C9. 

Milk, curd, and cheese were analysed at different steps of the process. Cheese samples were taken by boring holes which were then filled with sterile melted paraffin (Acofarma, Barcelona, Spain). Every sampling day, three samples were taken from each type of cheese and analysed by duplicate (n = 6). 

### 2.3. Cheesemaking Process

Four cheeses (~900 g per unit) were manufactured in a laboratory plant based on the typical process for Spanish hard cheese “Castellano”. This type of cheese is classified as hard and fat or extra fat. It is rennet coagulated and intensely pressed, with a firm and compact consistency, and a long shelf life. It is frequently manufactured from raw milk obtained in farms placed in Castilla y León, a region in the Northwest of Spain. The minimum aging period is 30 days for cheeses equal or under 1.5 kg in weight. Its final characteristics are 45% minimum fat in dry matter, 45% minimum dry matter, 25% minimum protein in dry matter, 3.26% maximum sodium chloride in dry matter, and pH value between 4.7 and 5.7.

For each cheese, 10 L of raw cow’s milk was heated (30 °C) and supplemented with 1.25 mL of calcium chloride (Laboratorios Arroyo, Santander, Spain). The starter culture was prepared following the manufacturer’s instructions. It included lyophilised strains of *Lactococcus lactis* subsp. *lactis*, *Lactococcus lactis* subsp. *cremoris*, *Lactococcus lactis* subsp. *lactis* biovar Diacetylactis, and *Streptococcus thermophilus* (CHOOZITTM MA 4001 LYO 5 DCU, Danisco, Sassenage, France). Temperature was increased up to 37 °C before the commercial starter culture was added (10 g/L). Milk for elaboration of spiked cheeses was inoculated with 100 mL of a 10^5^ cfu/mL working culture of each *E. coli* strain prepared as was described previously in Section 2.1. After 40 min, 3 mL of rennet (Laboratorios Arroyo, Santander, Spain, 1:10,000 strength) was added. The curd was cut and stirred until “rice grain” in size, heated to 38 °C, and transferred to moulds in order to be pressed at 0.01 kg/cm^2^ (1 h) and flipped for applying a second pressing (0.03 kg/cm^2^/5 h). The cheeses were salted by immersion in 20% (w/v) brine (7 °C/6 h) and kept at room temperature (20–24 °C) to drain for 24 h. Ripening was carried out at 10–12 °C and 85–90% relative humidity for 90 days.

At each cheesemaking step and throughout ripening, temperature was monitored using a Testo175-T2 (Instrumentos Testo S.A., Cabrils, Barcelona, Spain) data logger with internal sensor and external probe programmed to read every 2 min with an accuracy of 0.2 °C.

### 2.4. Microbiological Analysis

Samples from milk (10 mL), curd (10 g), and cheese after salting (10 g) were taken, homogenised in 90 mL of TSB-YE, and serially 10-fold diluted in peptone water solution. Twenty gram samples were taken during ripening and homogenised with 40 mL of TSB-YE to increase the detection limit of *E. coli* up to 2.5 cfu/g^-^ (0,40 log cfu/g). Homogenised samples in TSB-YE were incubated at 42 °C for 18 h to investigate the presence of the *stx* and *eae* genes by PCR.

For *E. coli* counts in milk, curd, and cheese, 1 mL of the appropriate dilutions was plated onto Tryptone Bile X- Glucuronide agar (TBX, Oxoid, Basingstoke, United Kingdom), and incubated at 37 °C for 24 h. In spiked cheese samples, white colonies (1.5–2 mm in diameter) were enumerated as aEPEC MK127C9 and blue colonies (1.5–2 mm in diameter) as STEC MK116C19. Five colonies from TBX plates from both control and spiked cheese samples were randomly tested by PCR for the presence of the *stx* and *eae* genes as indicated above. Moreover, Lactic Acid Bacteria (LAB) were counted using overlaid plates of de Man, Rogosa, and Sharpe Agar (MRS, Oxoid) incubated at 30 °C for 5 d.

### 2.5. Physicochemical Analysis

Determination of pH was performed with a Testo 205 pH meter (Testo Instruments, Lenzkirch, Germany) and water activity values (aw) were measured by using the AquaLab model CX-2 (Decagon, Pullman, WA, USA). NaCl concentration in cheese was determined as described in the AOAC Standard No 935.43 [31].

### 2.6. Statistical Analysis

Bacterial counts were transformed to log cfu per g (curd or cheese) or mL (milk). Physicochemical and microbiological values were statistically analysed for obtaining means and standard deviation. The Levene statistic was performed for testing the homogeneity of group variances. The effect of process stage on the investigated variables was carried out with one-way analysis of variance (ANOVA) and post hoc Tukey HSD test. The IBM SPSS Statistics for Windows, Version 26.0 (IBM Corp., Armonk, NY, USA) program was used for data analysis.

## 3. Results and Discussion

### 3.1. Physicochemical Parameters during Cheesemaking and Ripening

The physicochemical characteristics of the two different groups of cow’s cheese samples are listed in Table 1. 

The pH and aw values were decreasing throughout the cheesemaking, and the salt concentration was increasing in all the studied cheeses. At the beginning and the end of ripening, the physicochemical characteristics of cheese artificially contaminated with *E. coli* strains (pH, 4.99 ± 0.14 and 5.11 ± 0.04; aw, 0.96 ± 0.01 and 0.87 ± 0.01; NaCl, 2.60 ± 0.63% and 2.98 ± 0.37%) did not differ significantly (*p* > 0.05) from those found in control cheeses (pH, 5.19 ± 0.02 and 5.10 ± 0.09; aw_,_ 0.97 ± 0.01 and 0.88 ± 0.01; NaCl, 1.90 ± 0.42 and 3.25 ± 0.17). Therefore, both groups of hard cheeses were homologous according to these intrinsic factors and were within the typical range in similar commercial cheeses in Spain [32,33]. When studying the bacterial effectiveness of the whole process to obtain matured cheese, not only must the target microorganisms be considered, but also the interactive effects of a number of factors, some of them intrinsic parameters such as the ones determined herein. Even though *E. coli* belongs to the Enterobacteriaceae family and possesses important spoilage capabilities, intentional addition of this bacterium to the milk destined to cheesemaking makes no difference in terms of physicochemical characteristics of cheese. A few research works have addressed that survival of *E. coli* strains can be enhanced by cross-protection when subjected to combinations of stresses such as acid, salt and other factors [34]. It can be hypothesised that a synergistic effect of these different hurdle agents could be observed in cheese and that the sequence of application of different hurdles is important, but the majority of studies on growth / survival of STEC in cheese were not carried out with non-O157 serotypes [2].

### 3.2. Analysis of E. coli Populations in Cheese Samples

**Figure 1:**
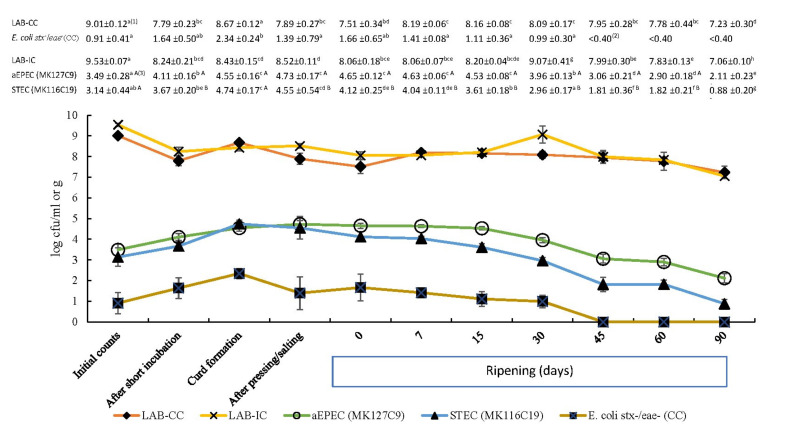
Evolution of indigenous *E. coli* (*stx*-/*eae*-) and lactic acid bacteria (LAB) counts in control cheese (CC), and aEPEC (MK127C9 strain), STEC (MK116C19 strain), and LAB counts in inoculated cheese (IC) during the manufacturing of raw cow’s milk cheese. Each displayed value shows the mean (log cfu/mL or log cfu/g) and standard deviation from six determinations. ^(1)^ In the same row, count means with different lowercase letters indicate statistical differences (*p* < 0.05) among cheesemaking and ripening steps. ^(2)^ Counts under the detection limit (<0.40 log cfu/g). ^(3)^ In the same column, means of STEC and aEPEC counts with different capital letters differ significantly (*p* < 0.05).

Milk used for the manufacture of cheese was negative for both *stx* and *eae* genes, thus free of STEC or EPEC contamination. Moreover, counts of *E. coli* on TBX-plates were ~1 log cfu/mL in the milk and were not above 2.34 log cfu/g (Figure 1) at their highest concentration (curd formation step) throughout the manufacturing process. Thus, this bacterial indicator was compliant with the hygienic requirements established in the Commission Regulation (EC) No 2073/2005 of 15 November 2005 [35]. Figure 1 shows the behaviour of bacterial populations during the cheesemaking process and cheese ripening (control and spiked samples). After inoculation of milk with both strains, counts were not significantly different (*p* > 0.05), reaching 3.14 ± 0.44 log cfu/mL for STEC MK116C19 and 3.49 ± 0.28 log cfu/mL for aEPEC MK127C9. STEC and aEPEC contamination of cow’s milk, though it is reported as ranging from 2 to 3% and ~6 to 10%, respectively [8], is not expected to reach the concentrations used in the spiked samples. These initial *E. coli* numbers would simulate a possible worst case scenario for food safety taking into consideration that contamination of milk with *E. coli* may certainly arise from milking to its final processing depending on how hygienic measures and manufacturing practices are carried out. The source of cheese contamination with foodborne pathogens may not only be raw milk, but also may occur during cheesemaking, aging, or storage from various sources, such as the operators, the plant environment, and the equipment [12]. It must be also highlighted that counts of STEC in cheese can be higher than usually reported, considering the occurrence of small groups of cells imbibed in the cheese matrix [27]. Moreover, low temperatures used during cheesemaking exert little inhibitory effect, in view of the fact that STEC strains appear to develop survival mechanisms for growing under chilled temperatures as was reported by Vidovic et al. [36].

The initial levels of the two tested strains steadily increased until the curd formation and a similar trend was observed on indigenous *E. coli* counts (*stx* -/*eae* -) in control cheese. All studied bacterial populations showed the highest counts during the curd formation after an increase of more than 1 log cfu/mL, in accordance with previously reported studies [22,23,24]. Thus, this initial multiplication is attributed to favourable growth conditions for *E. coli* at the beginning stages of cheesemaking, principally temperature (close to 37 °C), aw (around 0.99), and pH (greater than 5). Moreover, the significant increase in the studied pathogenic strains in the transition from milk (liquid) to curd (solid) can be mainly caused by bacterial concentration, owing to the fact that the curd behaves as a physical barrier trapping the bacteria and expelling the whey, concentrating up to ten times [24]. Thereafter, both autochthonous bacteria and the tested *E. coli* strains decreased their level throughout cheese aging.

### 3.3. Fate of aEPEC and STEC Strains in Raw Milk Cheese

**Figure 2:**
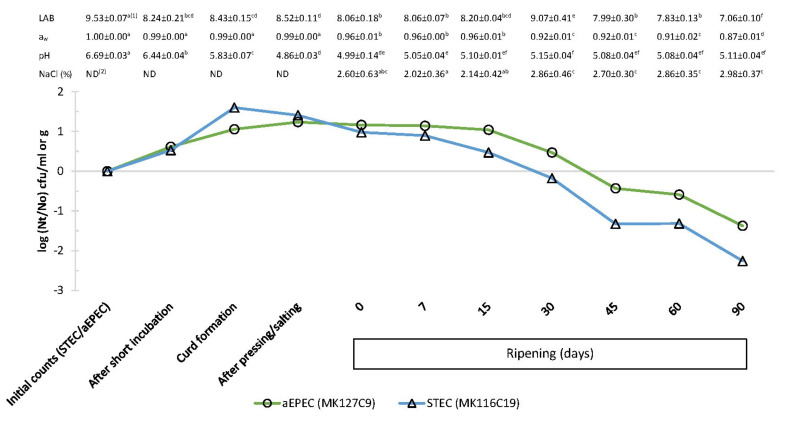
Effect of cheesemaking and ripening on aEPEC (MK127C9 strain) and STEC (MK116C19 strain) inoculated (3.1–3.5 log cfu/g) in raw cow’s milk cheese, and lactic acid bacteria (LAB) counts (log cfu/mL or log cfu/g), water activity (aw), pH, and NaCl at the same sampling points. Each displayed value shows the mean and standard deviation from six determinations. ^1^ In the same row, means with different lowercase letter indicate statistical differences (*p* <0.05) among cheesemaking and ripening steps. ^2^ ND, not determined.

A different behaviour between the diarrhoeagenic *E. coli* strains was noted after cheese pressing, with STEC counts decreasing significantly faster than aEPEC strain counts (*p* < 0.05). From that point, and during the cheese ripening, low pH, ranging from 4.99–5.15 (Table 1 and Figure 2), could be the factor explaining STEC behaviour. Oh et al. [37] found that, among several studied parameters in Cheddar cheese extracts, the low pH mainly affected the behaviour of STEC strains. It has been reported that although the optimum pH for STEC multiplication is around 7, it is capable of growing in a pH range between 4.5 and 9. Thus, these bacterial group find suitable pH for its multiplication in milk (pH 6–7) and also in foodstuffs of moderate acidity, such as some types of cheeses [15].

During the cheese maturation, the decrease in STEC and aEPEC populations was significant (*p* < 0.05) from day 7 and day 15, respectively, whereas indigenous *E. coli* population (*stx*-/*eae*-) on control cheese was below detection limit (<0.40 log cfu/g) after 30 d of ripening (Figure 1). The low pH was partly responsible for the reduction in aEPEC numbers. Nevertheless, our data suggest that a significant decrease in aw and the increase in salt concentration at the same time (Figure 2) acted as microbial hurdles in the cheese and could play an important role as well. It is assumed that the minimum aw required for *E. coli* multiplication is from 0.94 to 0.95, whereby it would be a limiting factor for the growth of this pathogen during the cheese ripening. Process control during ripening is equally important as controlling the cheesemaking process, as cheeses exposed to surface contamination when ripening can undergo changes in their physicochemical characteristics that may promote greater survival or even growth of *E. coli* [2]. Moreover, it has been reported that several factors during the manufacturing and cheese ripening, including NaCl concentration, acidity, storage temperature, and ripening point, may cause a synergistic effect on the growth of pathogenic *E. coli* [21,23].

The lactic acid bacteria (LAB) counts were around 8 log cfu/g during cheesemaking (Figure 1 and Figure 2), increasing their numbers in the curd and slightly decreasing throughout the ripening up to *ca.* 7 log cfu per gram. A similar trend was observed in the remaining investigated bacterial parameters. During the cheesemaking process of many varieties of cheeses, pH values drop to 5.2 caused by metabolic activity of LAB, being even sharper in some types [38]. LAB are mainly responsible for pH dropping, which, in accordance with our data, regularly declined until the beginning of ripening (Table 1). They also produce other substances by their natural activity that can inhibit bacterial growth, playing a key role in the control of pathogenic bacteria in cheese [39]. Natural microflora of raw milk as well as LAB used as starter cultures during cheesemaking can exert an antagonistic influence against STEC and other bacterial pathogens [40]. In our study, reduction in diarrhoeagenic *E. coli* numbers may be also related to LAB numbers for the reason that large initial LAB populations (up to ~8 log cfu/g) appear to be required to produce adverse effects on *E. coli*, particularly belonging to O157:H7 serotype [21]. However, there seems to be no compelling reason to argue for a strong antagonistic activity, by means of competition or antibiosis. Scientific literature reports both that *E. coli* was affected by indigenous microflora and not to play a significant role in its growth or survival [23].

Figure 2 shows that STEC population in inoculated cheese dropped below initial level (3.14 ± 0.44 log cfu/mL) after 30 days of ripening (−0.18 log (Nt/No) cfu/g). During the same period, the aEPEC strain kept its numbers above 3.49 log cfu/g (initial level of inoculum) and required 45 days of ripening to reduce the initial increase observed before maturation (−0.43 log (Nt/No) cfu/g). Regulation (EC) No 853/2004 of the European Parliament and of the Council of 29 April 2004 [41] introduced a ripening period of at least 60 days as a safety measure in cheeses manufactured from raw milk not complying with the microbiological criteria, which are established for total bacterial and somatic cells counts. Several studies, focused on pathogenic *E. coli* strains at different milk concentrations (10^1^–10^3^ cfu/mL) for cheese manufacturing, observed a significant reduction during 60 days of ripening, but none reported a complete elimination of this pathogen [21,22,23,24]. Our findings confirm these observations as, after 60 days of cheese ripening, the studied pathogenic *E. coli* populations decreased by 1.32 log cfu/g for STEC and by 0.59 log cfu/g for aEPEC compared to the corresponding bacterial levels inoculated in raw milk (Figure 2). These reduction ratios indicate that the 60-day aging requirement per se would be both ineffective to control completely aEPEC and uncertain for inactivating STEC. It is also noteworthy that both strains were still detectable after 90 days of ripening (Figure 1).

As far as we know, this is the first report showing the fate of an atypical EPEC strain during manufacturing of raw milk cheese. Our results point out that aEPEC O25:H2 (MK127C9 strain) adapted better than the STEC strain to the ripening conditions and reached higher numbers of survivals (2.11 ± 0.23 log cfu/g) after 90 days of maturation, showing an average reduction of less than 1.5 log cfu/g throughout the whole cheesemaking process. By contrast, STEC O140:H32 (MK116C9 strain) was more sensitive to ripening conditions but was also detected at the end of cheese maturation, determining a total reduction in counts lower than 2.5 log cycles. These facts suggest that when these pathogens, principally aEPEC, are in the raw milk, they could survive standard cheesemaking and remain viable in cheese beyond 60–90 days, as reported elsewhere for serotype O157:H7 [22,42]. The consideration of the serotype, beside the strain and type of cheese, is necessary to assess the bacterial effect of cheesemaking and ripening process in view of the fact that it was reported important differences on growth and persistence among STEC serotypes [22,23].

The main mechanisms involved in the survival of bacteria during cheesemaking are the acidic, osmotic, and heat shock stress responses, which can act individually or in combination. One of the key regulators of the general stress response in *E. coli* is the activation of the *rpoS* gene, which has been reported to differ among strains [38], thus being a possible explanation of the differences observed in our study. An additional explanation to the lower survival of STEC MK116C9 comparing with aEPEC MK127C9 may be due to the induction of Stx- encoding prophages. The different stresses produced during the cheesemaking and ripening could induce the lytic cycle [23,24] and, consequently, foster the reduction of STEC population. DNA of viral origin is a highly frequent element of the bacterial genome, which can be represented for fully functional prophages. These genetic elements can carry genes that influence the virulence of the bacterial host, such as Shiga toxin genes, or their metabolic activities. They can be activated spontaneously, merely as a result of randomness in gene expression or from induction of the host SOS response as it was reviewed by Nanda et al. [43]. This review set the focus on the triggering of spontaneous activity of prophages and the physiological consequences of this process on microbial populations. When prophage- carrying strains were grown in mixed populations, the spread of viral DNA was set, with a portion of bacteria being killed due to lytic development of prophages and survivors undergoing lysogenic conversion. Thus, the presence of lysogenic bacteriophages may add a potential drawback for survival of the bacteria, which is well used by bacterial competitors. The prophage- encoded Shiga toxin is a major virulence factor in Stx-producing *Escherichia coli*. Toxin production and phage production are linked and occur after induction of the RecA-dependent SOS response. This induction could be promoted by food- related stress. Fang et al. [44] observed that the effect of stressors, such as lactic acid or sodium chloride, reduced bacterial counts by 1 to 2 log cfu/mL in some foods.

## 4. Conclusions

Pathogenic *E. coli* inactivation during the cheesemaking process is conditioned not only by strain, but also by pathotype. Our results show that the first steps of cheesemaking allow for an increment in STEC and aEPEC counts and that STEC is more sensitive to the ripening than aEPEC. A moderate contamination by these *E. coli* pathotypes on cheese manufactured from raw milk may not be totally controlled through the process. Thus, low concentrations (10^1^–10^2^ ufc/mL) of these diarrhoeagenic *E. coli* strains in milk may finally result in a significant level of these pathogens in cheese after an ageing period of more than 60 days.

It seems necessary to guarantee the best bacteriological quality of raw milk intended for cheese through application of good farming practices in order to prevent cross-contamination and, thus, to reduce the risk of pathogenic *E. coli* in the dairy industry.

## Figures and Tables

**Table 1:** Changes in pH, aw, and salt during the manufacturing and ripening of raw cow’s milk cheeses.

Steps of Manufacture	Control Cheese ^1^			Inoculated Cheese ^2^		
	pH	aw	NaCl	pH	aw	NaCl
Milk	6.76 ± 0.01^a 3^	1.00 ± 0.00 ^a^	ND ^4^	6.70 ± 0.03 ^a^	1.00 ± 0.00 ^a^	ND
After short incubation	6.44 ± 0.03 ^b^	1.00 ± 0.00 ^a^	ND	6.44 ± 0.04 ^b^	0.99 ± 0.00 ^a^	ND
Freshly cut curd	5.67 ± 0.06 ^c^	0.99 ± 0.00 ^ab^	ND	5.83 ± 0.07 ^c^	0.99 ± 0.00 ^a^	ND
Curd after moulding	5.00 ± 0.09 ^d^	0.99 ± 0.00 ^ab^	ND	4.86 ± 0.03 ^f^	0.99 ± 0.00 ^a^	ND
After pressing/salting (0 d)	5.19 ± 0.02 ^g^	0.97 ± 0.01 ^bc^	1.90 ± 0.42 ^a^	4.99 ± 0.14 ^e^	0.96 ± 0.01 ^b^	2.60 ± 0.63 ^abc^
Cheese ripening:						
7 d	5.08 ± 0.02 ^def^	0.96 ± 0.01 ^c^	2.45 ± 0.66 ^ab^	5.05 ± 0.04 ^de^	0.96 ± 0.00 ^b^	2.02 ± 0.36 ^a^
15 d	5.13 ± 0.04 ^fg^	0.95 ± 0.01 ^cd^	2.66 ± 0.60 ^abc^	5.10 ± 0.01 ^d^	0.96 ± 0.01 ^b^	2.14 ± 0.42 ^ab^
30 d	5.04 ± 0.04 ^def^	0.93 ± 0.02 ^de^	2.96 ± 0.56 ^bc^	5.15 ± 0.04 ^d^	0.92 ± 0.01 ^c^	2.86 ± 0.46 ^c^
45 d	5.02 ± 0.05 ^de^	0.92 ± 0.01 ^e^	3.26 ± 0.19 ^c^	5.08 ± 0.03 ^de^	0.92 ± 0.01 ^cd^	2.70 ± 0.30b ^c^
60 d	5.12 ± 0.07 ^efg^	0.91 ± 0.02 ^e^	3.02 ± 0.70 ^bc^	5.08 ± 0.04 ^de^	0.91 ± 0.02 ^d^	2.86 ± 0.35 ^c^
90 d	5.10 ± 0.09 ^defg^	0.88 ± 0.01 ^f^	3.25 ± 0.17 ^c^	5.11 ± 0.04 ^d^	0.87 ± 0.01 ^e^	2.98 ± 0.37^c^

^1^ Cheese manufactured from cow’s milk. ^2^ Cheese manufactured from cow’s milk spiked with diarrhoeagenic *E. coli* strains: STEC MK116C19 (O140:H32 serotype) and aEPEC MK127C9 (ONT:H2 serotype). ^3^ In each column, means bearing different lowercase letters differ significantly (*p* < 0.05; n = 6). ^4^ ND, not determined.
